# Stroke Rehabilitation Reaches a Threshold

**DOI:** 10.1371/journal.pcbi.1000133

**Published:** 2008-08-22

**Authors:** Cheol E. Han, Michael A. Arbib, Nicolas Schweighofer

**Affiliations:** 1 Department of Computer Science, University of Southern California, Los Angeles, California, United States of America; 2 USC Brain Project, University of Southern California, Los Angeles, California, United States of America; 3 Department of Computer Science, University of Southern California, Los Angeles, California, United States of America; 4 Department of Neuroscience, University of Southern California, Los Angeles, California, United States of America; 5 Department of Biokinesiology and Physical Therapy, University of Southern California, Los Angeles, California, United States of America; University College London, United Kingdom

## Abstract

Motor training with the upper limb affected by stroke partially reverses the loss of cortical representation after lesion and has been proposed to increase spontaneous arm use. Moreover, repeated attempts to use the affected hand in daily activities create a form of practice that can potentially lead to further improvement in motor performance. We thus hypothesized that if motor retraining after stroke increases spontaneous arm use sufficiently, then the patient will enter a virtuous circle in which spontaneous arm use and motor performance reinforce each other. In contrast, if the dose of therapy is not sufficient to bring spontaneous use above threshold, then performance will not increase and the patient will further develop compensatory strategies with the less affected hand. To refine this hypothesis, we developed a computational model of bilateral hand use in arm reaching to study the interactions between adaptive decision making and motor relearning after motor cortex lesion. The model contains a left and a right motor cortex, each controlling the opposite arm, and a single action choice module. The action choice module learns, via reinforcement learning, the value of using each arm for reaching in specific directions. Each motor cortex uses a neural population code to specify the initial direction along which the contralateral hand moves towards a target. The motor cortex learns to minimize directional errors and to maximize neuronal activity for each movement. The derived learning rule accounts for the reversal of the loss of cortical representation after rehabilitation and the increase of this loss after stroke with insufficient rehabilitation. Further, our model exhibits nonlinear and bistable behavior: if natural recovery, motor training, or both, brings performance above a certain threshold, then training can be stopped, as the repeated spontaneous arm use provides a form of motor learning that further bootstraps performance and spontaneous use. Below this threshold, motor training is “in vain”: there is little spontaneous arm use after training, the model exhibits learned nonuse, and compensatory movements with the less affected hand are reinforced. By exploring the nonlinear dynamics of stroke recovery using a biologically plausible neural model that accounts for reversal of the loss of motor cortex representation following rehabilitation or the lack thereof, respectively, we can explain previously hard to reconcile data on spontaneous arm use in stroke recovery. Further, our threshold prediction could be tested with an adaptive train–wait–train paradigm: if spontaneous arm use has increased in the “wait” period, then the threshold has been reached, and rehabilitation can be stopped. If spontaneous arm use is still low or has decreased, then another bout of rehabilitation is to be provided.

## Author Summary

Stroke often leaves patients with predominantly unilateral functional limitations of the arm and hand. Although recovery of function after stroke is often achieved by compensatory use of the less affected limb, improving use of the more affected limb has been associated with increased quality of life. Here, we developed a biologically plausible model of bilateral reaching movements to investigate the mechanisms and conditions leading to effective rehabilitation. Our motor cortex model accounts for the experimental observation that motor training can reverse the loss of cortical representation due to lesion. Further, our model predicts that if spontaneous arm use is above a certain threshold, then training can be stopped, as the repeated spontaneous use provides a form of motor learning that further improves performance and spontaneous use. Below this threshold, training is “in vain,” and compensatory movements with the less affected hand are reinforced. Our model is a first step in the development of adaptive and cost-effective rehabilitation methods tailored to individuals poststroke.

## Introduction

Stroke is the leading cause of disability in the US, and about 65% of stroke survivors experience long-term upper extremity functional limitations [1]. Although patients may regain some motor functions in the months following stroke due to spontaneous recovery, stroke often leaves patients with predominantly unilateral motor impairments. Indeed, recovery of upper extremity function in more than half of patients after stroke with severe paresis is achieved solely by compensatory use of the less-affected limb [2]. Improving use of the more affected arm is important however, because difficulty to use this arm in daily tasks has been associated with reduced quality of life [3].

There is now definite evidence however that physical therapy interventions targeted at the more affected arm can improve both the amount of spontaneous arm use and arm and hand function after stroke [4]. Further, even after motor retraining is terminated, performance can further improve in patients with less severe strokes in the months following therapy [5],[6]. A possible interpretation of this result is that the repeated attempts to use the affected arm in daily activities are a form of motor practice that can lead to further improvements in motor performance [5].

The neural correlates of motor training after stroke have been investigated in animals with motor cortex lesions [7],[8]. Specifically, a focal infarct within the hand region of the primary motor cortex causes a loss of hand representations that extends beyond the infarction. However, several weeks of rehabilitative training can overcome this loss of representation, and yield an expansion of the hand area to its prelesion size; the larger area in turn has been correlated with higher level of performance [9]. Long-term potentiation in pyramidal neuron to pyramidal neuron synapses has been demonstrated in horizontal lateral connections [10], and may provide the basis for map formation and reorganization in the motor cortex [11], and motor skill learning [10].

Contrasting with the increase in performance due to spontaneous recovery, a concurrent *decrease* of spontaneous arm use has been proposed to occur following stroke. This decrease may be due both to the higher effort and attention required for successful use of the impaired hand and to the development of *learned nonuse* [12], in that the preference for the less affected arm is learned as a result of unsuccessful repeated attempts in using the affected arm [13]–[15]. The constraint-induced therapy (CIT) protocol, which forces the use of the affected limb by restraining the use of the less affected limb with a mitt, has been specifically developed to reverse learned nonuse [16]. Although its “active ingredients” are still not well understood [17], CIT has been shown to be effective in the recovery of arm and hand functions after stroke in multisite randomized clinical trials [4]. Because 50% of the eventual improvement in use (as measured by the questionnaire-based “motor activity log”) is seen at the end of the first day of CIT, it has been suggested that CIT is effective in reversing learned nonuse [18]. To our knowledge, however, there are no longitudinal data tracking the development of learned nonuse just after stroke and during recovery.

In summary, increase in performance after stroke due to spontaneous recovery, rehabilitation, or both does not appear to correlate simply with spontaneous arm use, and a yet-to-be clarified nonlinear mechanism seems to be at play. Here, we focus on rehabilitation in the control of reaching poststroke, a prerequisite for successful manipulation. We developed a biologically plausible model of bilateral control of reaching movements to investigate the mechanisms and conditions leading to such positive or negative changes in spontaneous choice of which arm to use. Our central hypothesis, based on the above observations, is the existence of a threshold in spontaneous arm use: if retraining after brain lesion (or spontaneous recovery) increases spontaneous arm use above this threshold, performance will keep increasing, as each attempt to use the affected arm will act as a form of motor relearning. The patient will then enter a virtuous circle of improved performance and spontaneous use of the affected arm, and therapy can be terminated. In contrast, if spontaneous use of the arm does not reach this threshold after either natural recovery or rehabilitation, or both, performance will not improve after stroke, and compensatory strategies with greater reliance on the less affected arm will either remain or even develop further.

## Methods

### Behavioral Setup

**Figure 1:**
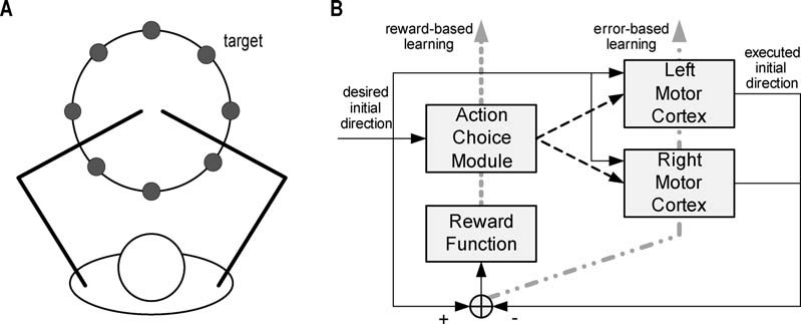
Experimental setup and model structure. (A) Experimental setup. (B) Model structure. Solid line: information signal; dashed line: activation signal; dotted line: reward-based (reinforcement) learning; double dotted line: error-based (supervised) learning.

To model spontaneous use of one arm or the other, and changes in motor performance, we simulated horizontal reaching movements towards targets distributed along a circle centered on the initial (overlapping) positions of the two arms (Figure 1A). Our computational model of bilateral arm use in arm reaching contains a left and a right motor cortex, and a single action choice module (Figure 1B). We first trained the full model (the “normal subject”) to reach with either hand, but with a bias for using the hand closer to the eventual target. Spontaneous arm use was recorded in a free choice condition, in which the action choice module can select either arm to reach targets that are randomly generated anywhere along the circle. Motor performance was evaluated by the directional error between the desired movement direction and the actual hand direction.

To simulate stroke, we partly lesion one hemisphere (i.e., remove a set of simulated neurons from the simulation). We first simulate a spontaneous recovery period in which the action choice module determines the choice of arm, and the state of motor cortex determines error in reaching, with consequent changes in synaptic weights. We then mimic CIT with a forced use condition in which only the use of the affected arm (i.e., that contralateral to the lesioned cortex) was allowed. We study in simulations the conditions that lead to successful recovery, that is, to high levels of spontaneous use and performance with the affected arm in appropriate regions of space, and low reliance on compensatory movements with the less affected arm.

### Computational Model

Our model has two distributed interacting and adaptive systems: the motor cortex for motor execution and the action choice module for decision-making.

#### Motor cortex model

We made two assumptions to model the motor cortex with a left and a right module for control of the contralateral arm:

1. The motor cortex contains neurons coding direction of hand movement [19] with signal dependent noise [20],[21]. Although the issue of correlation versus coding for hand directions is a subject of intense debate [22]–[25], computational models have developed the view that motor cortex neurons linked to arm muscles exhibit activity strongly correlated with hand direction in the initial phase of the movement [26],[27]. This assumption allowed us to simplify the model considerably by not requiring us to model a spinal cord, muscles, and arms linking the output of the motor cortex to the behavior.

The activation rule of each motor neuron is given by a truncated cosine function [28] based on the empirical data of [19] which correlates the firing rate of neuron *i* with the difference between the “preferred direction” *θ_p_^i^* (that associated with maximal firing of this neuron) and the currently chosen hand direction, *θ_d_*:
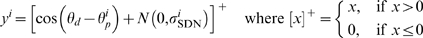
(1)where *y^i^* is the firing rate of the *i*th neuron. *N*(0,*σ^i^*_SDN_) is normally distributed signal dependent noise with zero mean and standard deviation proportional to the mean signal size [20], [21], [28]–[30], that is, 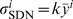 where *y̅* *^i^* is the noiseless activation 
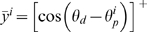
.

Summation of individual neuron vectors (with each vector length given by Equation 1, and the vector direction given by the preferred direction) yields a population vector that has been shown to be well aligned with the initial actual (executed) hand direction *θ_e_* [19]. In our model, at each action, one half (left or right) of motor cortex is chosen to control the next reaching movement (see below). Thus, we take the actual reaching direction to be that given by the direction of the population vector of the chosen motor hemicortex.

1. The motor system learns to generate reaching movements by minimizing error bias and by recruiting more neurons for frequently used movement, in effect minimizing directional variance [21]. We now specify how neurons' preferred directions in the active hemisphere are slowly modified after each trial. Mathematically, we view a learning rule as an adjustment of parameters that serves to improve the performance of the system with respect to some criterion. As we shall see below, such learning is not always best for other behavioral criteria. For the motor cortex, we measure performance with the following cost function, which is a function of reaching error and total neuronal activity:
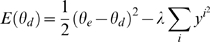
(2)where *θ_d_* is the desired direction, *θ_e_* is the direction specified by the population vector of the motor cortex (a function of the synaptic weights therein), and *λ* is a free parameter. The first term of the right hand side of Equation 2 measures the directional error, and the second part the total neural activity, which is related to the magnitude of the population vector.

The cost can, with some approximation, be decreased by applying the following motor cortex learning rule (Text S1):
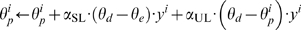
(3)where *α*_SL_ and *α*_UL_ are learning rates. The first term of the learning rule, a supervised learning term that resembles a standard supervised learning rule in linear neurons [31], decreases the global directional error. Support for this term of the rule stems from monkey experiments, in which adaptation to an external force field or to visuo-motor rotations induces neuronal reorganization of preferred direction in primary motor cortex neurons [32],[33]. The second term of the learning rule, an unsupervised learning term that resembles the standard unsupervised competitive learning rule [31], orients the neurons' preferred directions towards the desired reaching direction.

#### Action choice module

In reinforcement learning, actions that maximize outcomes are selected based on estimates of future cumulative rewards, or “values” [34]. Reinforcement learning provides a plausible framework for human adaptive decision-making with desirable theoretical and biological properties, [35]–[37]. There is evidence that values are acquired by cortico-basal ganglia networks [35],[38],[39], under the influence of the dopaminergic system [40],[41]. Further, it is likely that basal ganglia output releases inhibition of the motor cortex for selected actions [42]. Our action choice module (Figure 1B) thus utilizes reinforcement learning to learn how to choose which arm to use in reaching each target based on a comparison of the values of using one arm or the other. Such “action” values have been recently shown to be represented in the striatum [35]. The action values are learned from the reward prediction error *δ*, the difference between the actual reward, which evaluates the executed action, and the predicted reward, as estimated by the action value [34]. We now turn to the definition of these quantities.

Here, we use a total (internal) reward *r*_total_ with two components: First, healthy subjects tend to use the left arm to reach to the left, and similarly for the right, but with a handedness preference near the midline [43]. As each subject's level of comfort correlates with arm use [43], we model workspace preference of hand with a reward term that is positive if the right arm is used in the right hand side workspace (RHS) or the left arm is used in the left hand side workspace (LHS). Second, we use a performance-related reward term, which is high when the executed direction *θ_e_* is close to the given desired direction *θ_d_* and low if the direction of the actual movement deviates from the desired direction. The total reward is thus given by:
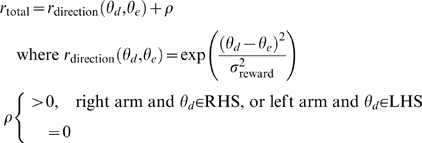
(4)where *σ*_reward_ is the broadness of the reward function and *ρ* gives the workspace preference of the hand.

The action choice module selects one of the arms for movement execution by comparing the action values *Q*(*a_i_*, *θ_d_*), that is, the reward expected by selecting arm *a_i_* for the desired direction *θ_d_*, with *a_i_*∈[left, right] and *θ_d_*∈[0,360°]. Although a number of function approximators can be used to learn the action values, our results are not dependent on the exact choice of approximators. Here we used two radial basis function (RBF) networks to estimate the action values, one for each of the two possible actions. RBF is a form of linear regression with exponential basis functions; the estimated values are thus computed with:
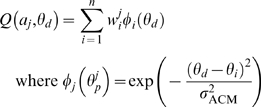
(5)where *Q* is the estimated action value, *w_i_^j^* are tunable weights for action *a*, *n* is the number of RBFs, *θ_i_* is the center of the *i*th RBF, and *σ*_ACM_ is the broadness of each RBF, which is chosen to be equal to *π*/*n* as this allows good generalization [44].

After each movement, the action value of each arm is updated with the reward prediction error, that is, the difference *δ* = *r*_total_−*Q*(*a*, *θ_d_*) between the actual reward and the expected reward. The weights *w_i_^a^* are updated to minimize the square of the reward prediction error *δ* ^2^.
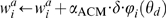
(6)where *α*_ACM_ is a learning rate.

Based on the action values, the module probabilistically selects which motor cortex will be used to execute a movement according to the softmax function [34]:
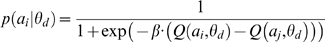
(7)where the parameter *β* controls the variability of action choice, with a large *β* yielding less variability, *a_i_*∈[left, right] and *θ_d_*∈[0,360°].

### Simulations

**Figure 2:**
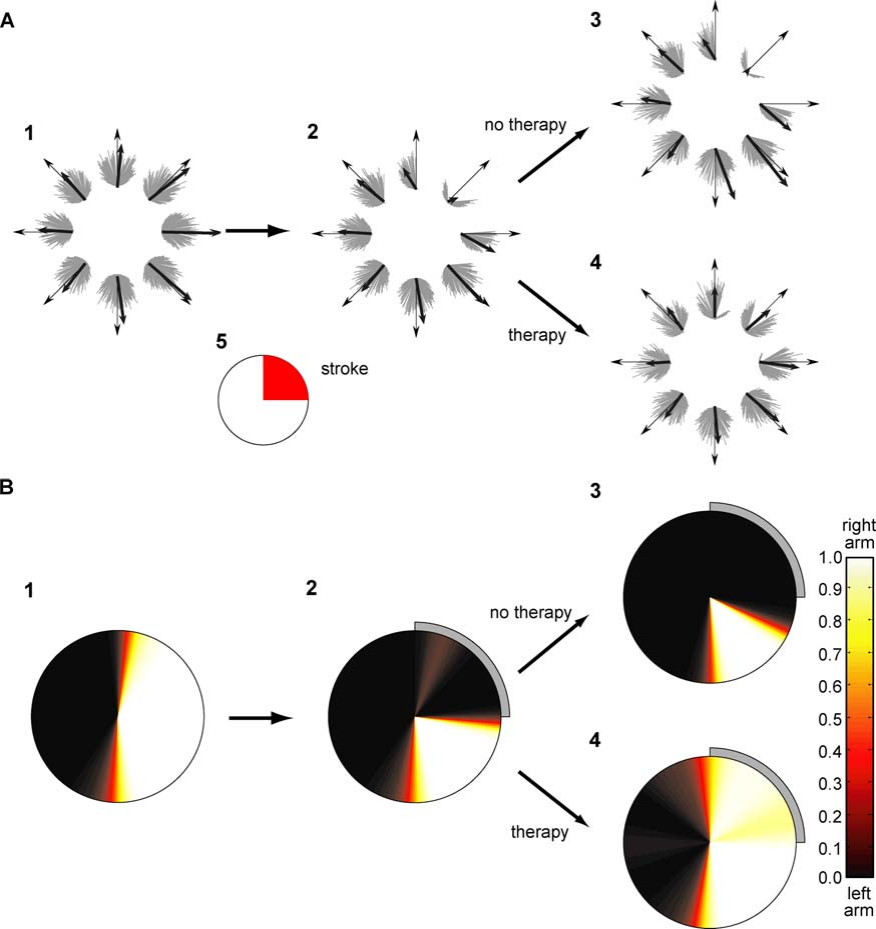
Neuronal population coding and spontaneous use over the workspace for the affected arm. (A) Neuronal population coding. (B) spontaneous use (B). For (A) and (B): (1) Before stroke, (2) after stroke, (3) after 3,000 free choice trials, and (4) after 1,000 forced used trials followed by 2,000 free choice trials. In (A), each population vector figure shows the desired reach directions (thin black arrows), the neuron activation levels along their preferred directions (thin gray lines), and the resulting population vector (thick black arrows). Note that there are no “votes” for directions corresponding to the lesioned directions in (A.2) and (A.3) but that in (A.4), many neurons have become retuned to yield votes in the lesioned directions. In (B), the pie plots show the probability of using the unaffected right arm to reach to targets arrayed on a circle around the central position. In (B.2) and (B.3), the less affected arm reaches into the lesioned quadrant, but this effect is reversed with therapy (B.4).

Strokes seem to affect only a certain range of movement directions. Outside this range, reaching is relatively spared [45]. To model this effect, we removed the neurons with preferred directions in the first quadrant of the left motor cortex (50% of the neural population coding for the right hand side workspace, as shown in Figure 2A.5), which controls the right arm (unless otherwise noted). The results would be the same had we chosen the other arm, or any other quadrant. We also tested stroke models in which neurons were affected probabilistically as a function of the range angle (with neurons being removed with 100% probability for the central angle of the simulated lesion and then with lower probability as the angles on each side of the lesion center increase); simulation results with these stroke models were qualitatively similar to those with the “hard boundary” model and thus for simplicity are not presented here. We also tested different stroke patterns, including a lesion ranging from 45° to 145°, and lesions with asymmetric bimodal distributions. Simulations (results not shown) confirmed that such lesions did not produce results qualitatively different from those presented here.

We used two measures of motor performance:

1. The absolute value of the directional error between the intended reach direction and the population vector direction.
2. The magnitude of the population vector, normalized by the magnitude of the population vector before stroke.

We chose these two performance measures in our model because they can be linked to actual patient performance measures. Initial directional error has been used in characterizing reaching in stroke patients (e.g., [46]). Although the population vector is normally not directly observable in patients, it can be regarded as a measure of force exerted by arm muscles on the hand [26],[47],[48], and low force generation is a characteristics of stroke [49]. Because both use and performance are stochastic, we report averages of 10 uniformly distributed samples over the affected range in all graphs (except the pie charts of Figures 2, 8, and 9).

The changes in performance and spontaneous arm use of the affected arm were recorded in four consecutive phases: (i) an acquisition phase of normal bilateral reaching behavior in 2,000 free choice trials (partially shown), (ii) an acute stroke phase of 500 free choice trials, (iii) a rehabilitation phase in a forced use condition (variable number of trials), and (iv) a chronic stroke phase consisting of 3,000 free choice trials. Values of performance and spontaneous use just after rehabilitation are called “immediate;” their long-term values at the end of the chronic phase are called “follow-up.”

In all phases, targets were randomly generated at the start of each trial, distributed uniformly across all possible angles. Unless otherwise stated, we used the following parameters: Each motor cortex had 500 neurons, with initial preferred directions *θ_p_* uniformly distributed. The coefficient of variation of the signal-dependent noise ratio *k* was 0.15. The motor cortex learning rates were *α*_SL_ = 0.005 and *α*_UL_ = 0.002. The action choice module contained two networks of 20 radial basis function neurons with *σ*_reward_ = 0.2 (in radians, ≈11.46°), *ρ* = 0.2, *σ*_ACM_ = *π*/10 (in radians,  = 18°), *α*_ACM_ = 0.1, and *β* = 10.

## Results

The first (prelesion) phase provided a normal baseline for reaching behavior. For each desired direction, learning achieved zero mean directional error (Figure 2A.1) and a tendency of right arm use for the right-hand-side workspace, and left arm use for the left-hand-side workspace (Figure 2B.1).

Just after stroke, however, the population vectors showed directional errors in and around the affected range (Figure 2A.2). Sufficient therapy (1,000 forced use trials, Figure 2A.4) resulted in redistributing the preferred directions within the affected side of motor cortex, with the population vectors realigned to the desired directions. Although the realignment was not perfect, and a small range of preferred directions was still missing, the directional errors were much reduced. This resulted in increased rewards in these directions, thus increasing the action value for the affected arm, preparing the way for increased use of the affected arm once free choice was allowed. Lack of therapy on the contrary resulted in a still large missing range of directions (Figure 2A.3).

At the end of the “acute stroke” period, the less affected arm largely compensated for the more affected arm in the affected range (Figure 2B.2). If no therapy followed, then this behavioral compensation remained (Figure 2B.3). Sufficient therapy, however, led on the resumption of free choice trials to increased spontaneous arm use of the more affected arm (right arm) in the affected range (Figure 2B.4) and almost restored it to its prestroke levels.

**Figure 3:**
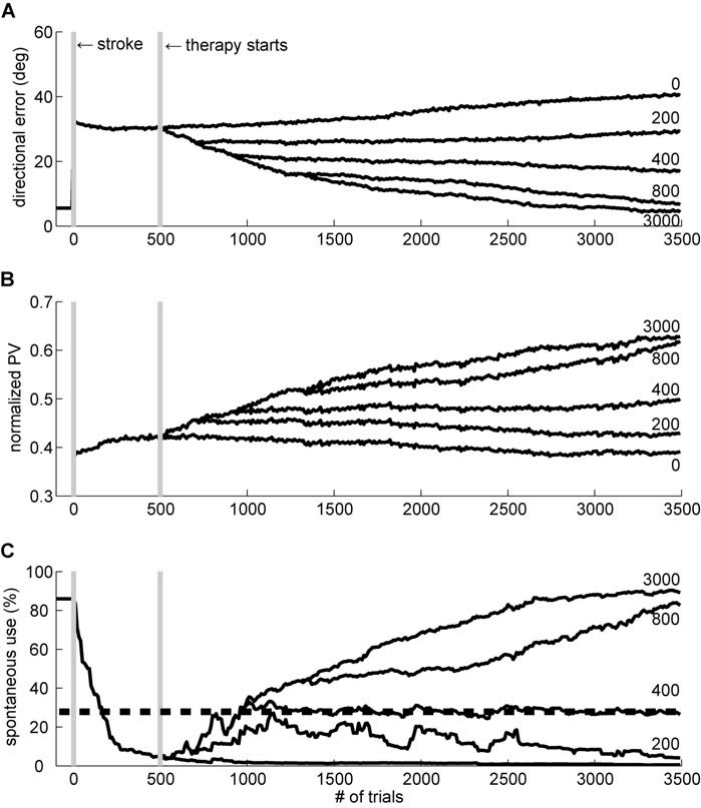
Time course of behavioral performance and spontaneous use in the affected range just before stroke, following stroke (“acute stroke”), during rehabilitation, and after rehabilitation (“chronic stroke”). (A) Directional error, (B) normalized population vector (PV), and (C) spontaneous arm choice. Five different durations of therapy were used (0, 200, 400, 800, or 3,000 trials). The spontaneous arm use is an average selection probability from 10 uniformly distributed desired directions on the affected range. The threshold of effective rehabilitation for this stroke size is shown in the horizontal dotted line of (C). If the rehabilitation leads to performance above this threshold, then a virtuous circle between spontaneous arm use and performance will take place and performances will continue to improve without the need for further rehabilitation.

We then studied the time courses of motor performance measures and spontaneous arm use (Figure 3). In the acute stroke phase, the free choice condition resulted in some spontaneous recovery in performance, as the repeated attempts to use the arm, although generated with poor performance, produced directional errors that retuned the motor cortex. However, the poor performance of these initial repeated attempts to use the affected arm caused a decrease in the action value for this arm in the affected directions, leading in turn to a reduction in spontaneous arm use. Thus, a “learned nonuse” phenomenon occurred despite improving performance. After 500 trials of natural recovery, a number of rehabilitation trials were given in the forced use condition. Rehabilitation improved performance as expected, but its lasting effects on spontaneous arm choice depended on the intensity of therapy. The increase in spontaneous arm use returned close to 0% soon after the end of therapy if only 200 trials of therapy were given. If 400 trials of therapy were given, spontaneous arm use held steady after therapy. If more therapy was given, spontaneous arm use was high after therapy and kept improving for a large number of trials thereafter.

**Figure 4:**
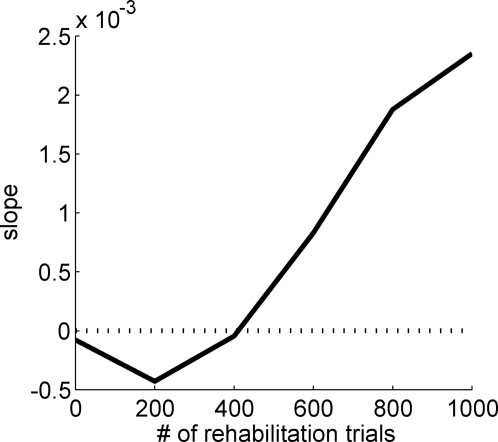
Changes in spontaneous use following rehabilitation as a function of the number of rehabilitation trials. We plotted the average slope of spontaneous arm use in the 1,000 trials following rehabilitation as a function of the intensity of therapy. Above 420 trials (with the default parameter set), spontaneous arm use increases after therapy. Below this number of trials, it decreases.

**Figure 5:**
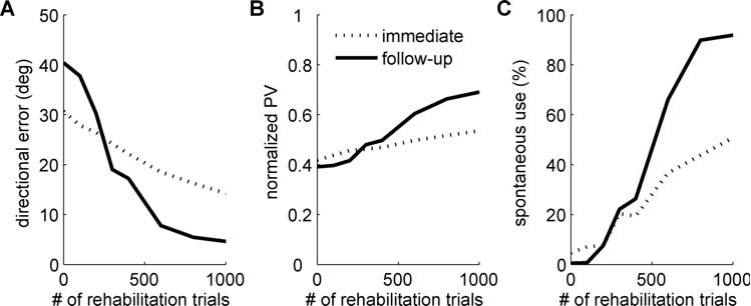
Effect of the number of rehabilitation trials in immediate and follow-up tests. Directional error (A), normalized population vector (PV) (B), and spontaneous arm use (C) in the immediate and follow-up tests. The directional error performance following few rehabilitation trials worsens after therapy. On the contrary, the directional error performance after sufficient rehabilitation trials improves even after therapy. Similar bistable patterns are shown for the normalized population vector and spontaneous use shown in (B) and (C).

The model thus exhibits a threshold for the intensity of rehabilitation. To precisely quantify the threshold, we computed the change in spontaneous arm use following rehabilitation by fitting a simple linear model with trials post stroke as predictor; the number of trials corresponding to a null slope corresponds to this threshold. As shown in Figure 4, with the default parameter set, there was a threshold at 420 trials of forced used trials, above which spontaneous arm use increased even after therapy was discontinued. Below this number of forced used trials, spontaneous arm use decreased to minimal levels after rehabilitation—it was “in vain.” The zero crossing in the slope in Figure 4 implies bistability of spontaneous arm use: when the number of rehabilitation trials is larger than the number of trials required to reach the threshold (420 trials), the spontaneous arm use improves in the following free choice condition until it saturates; conversely, when the number of therapy rehabilitation is less than the number of trials required to reach the threshold, the spontaneous arm use deteriorates (Figure 5C). Similar bistability is also shown in the directional error (Figure 5A) and normalized population vector (Figure 5B).

**Figure 6:**
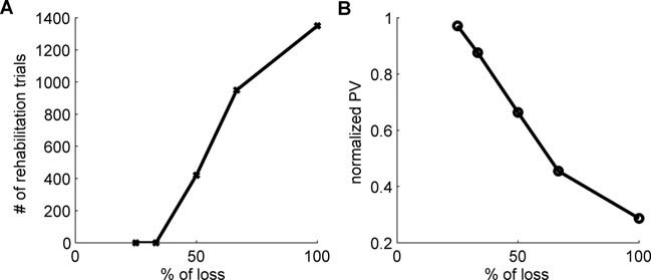
Effect of stroke size. (A) Number of rehabilitation trials required to reach the effective rehabilitation threshold, as a function of lesion sizes. (B) Normalized population vector (PV) as a function of lesion size in the follow-up test after 800 rehabilitation trials.

As expected, the minimal intensity of effective therapy depends on lesion size (Figure 6A). Compared to smaller lesions, large lesions require longer rehabilitation sessions to reach the threshold of spontaneous arm use above which therapy can be terminated. In our model, although directional error recovered almost perfectly for lesions sizes smaller than 50% for the right hand side workspace (follow-up test after 800 rehabilitation trials; results not shown), the long-term normalized population vector correlates almost linearly to the lesion size (same simulations conditions, see Figure 6B).

**Figure 7:**
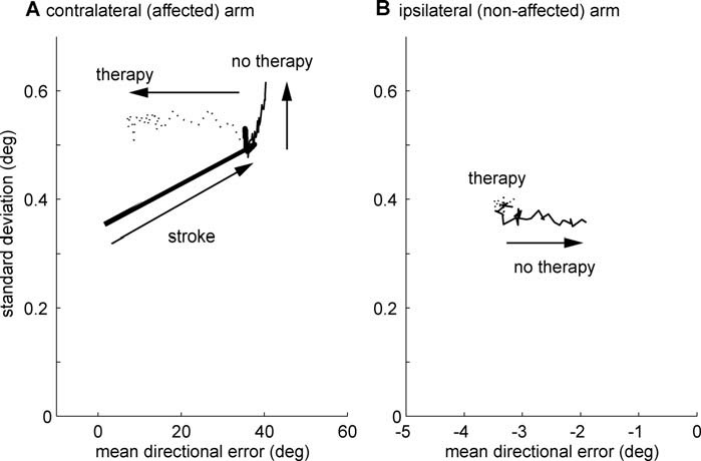
Changes in reach precision (standard deviation of directional error) in relation to changes in accuracy (mean of directional error). (A) Contralateral (affected) arm and (B) ipsilateral (nonaffected) arm. In each panel, the thick solid line corresponds to the changes occurring from just before stroke to the 500th free choice trials following stroke onset. The thin solid line represents additional changes in a no therapy condition (3,000 free choice trials). The dotted line represents additional changes in a therapy condition (1,000 therapy trials followed by 2,000 free choice trials). After stroke, accuracy and variability of the contralateral arm worsened. Following therapy, accuracy improved but with little change in variability. With no therapy, behavioral compensation with the nonaffected arm further developed, resulting in improved accuracy for this arm (B).

Motor performance can be judged according to two different criteria: accuracy (low bias of error) and precision (low variance of error). Figure 7 shows the effects of stroke and therapy, or the lack of it (‘no therapy’), on the accuracy and precision of the reach directional error over the affected range for the affected arm (contralateral to the lesion, Figure 7A) and for the nonaffected arm (ipsilateral to the lesion; Figure 7B). Although, stroke leads to an immediate and large deterioration of accuracy and precision for reaching movements with the affected arm (Figure 7A, thick solid line), therapy restores accuracy to near prestroke level (Figures 7A, dotted line). Because the number of available neurons is reduced after stroke, however, precision remains low after therapy compared to prestroke levels (Figure 7A). Lack of therapy (‘no therapy’ in Figure 7A, thin solid line) results in further deterioration of accuracy and precision for the affected (right) arm after stroke. In contrast, while stroke and therapy have almost no effect on performance of the nonaffected arm in our model (Figure 7B, dotted line), the increased frequency of compensatory reaching movements in the no therapy condition results in an increase of accuracy on these reaching movements (Figure 7B, thin solid line).

**Figure 8:**
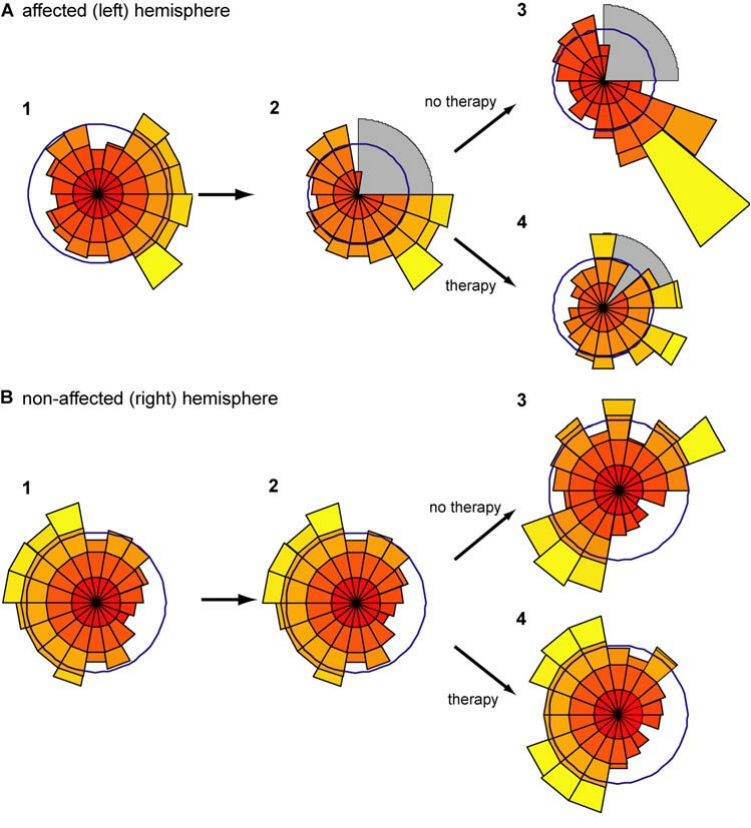
Cortical reorganization following stroke. Reorganization of the affected (left) hemisphere (A) and nonaffected (right) hemisphere (B) after stroke followed by therapy or no therapy. In each panel, histograms of the cells' preferred directions are shown (1) before stroke, (2) after stroke with 500 free choice trials, and (3) after 3,000 free choice trials or (4) after 1,000 forced used training trials and subsequent 2,000 free choice trials. The gray area in (A.2) shows the lesion site. Before the lesion, the left hemisphere contains more neurons with preferred directions in the right workspace, and the right hemisphere contains more neurons for the left workspace because of the bias for workspace preference. Just after lesion, the left hemisphere is affected. If no therapy follows, the size of the affected range increases, and the number of neurons for the fourth quadrant increases in the affected hemisphere (maladaptation) and in the first quadrant in the nonaffected hemispheres (A.3). On the contrary, the number of neurons for the first quadrant in the right hemisphere increases due to compensation. After therapy (1,000 forced use trials followed by 2,000 free choice trials), however, the distributions of directions are similar to the prelesion distribution in both hemispheres.

We then studied the organization and reorganization of the cells' preferred directions in each hemisphere before lesion, after lesion, and after therapy. Using pie histograms (Figure 8) which show the number of neurons whose preferred directions are in a certain range of directions, we observed a cortical reorganization pattern similar to that observed in animals that undergo rehabilitation or not after motor cortex lesions (see Discussion). Before lesion, more cells coded for the movements that were more often performed. After lesion, therapy or the lack of it affects the reorganization of neurons' preferred directions in both hemispheres.

### Therapy

Motor training with the affected arm has a profound effect on reorganization in the affected hemisphere. After sufficient therapy, the distribution of the surviving cells' preferred directions is similar to the prelesion distribution, with, however, fewer cells coding each direction, because the total number of cells is reduced (Figure 8A.4). During therapy, the directional error decreases, ensuring concordance of the supervised and unsupervised learning rules; the unsupervised learning rule is “adaptive” as it reinforces the supervised learning rule (Figure 8A.4). Conversely, motor training has almost no effect on the cell population of the nonaffected arm (Figure 8B.4).

### No therapy

Two patterns of reorganization are noteworthy in the affected hemisphere. First, the size of the affected range increased compared to just after the lesion; second, a large number of cells now code for movements in the fourth quadrant. If no therapy or insufficient therapy is provided, the directional error of the affected arm does not decrease (Figures 3A and 7A). This results in discordance between the supervised and unsupervised learning rules, and the unsupervised learning rule, based on desired but not actual directions, becomes “maladaptive,” further increasing the lesion size (Figure 8A.3) and largely increasing the representation of compensatory movements (Figure 8B.3) whose performance improves (decrease both in directional error bias and in directional error variability, and increase in normalized population vector). In the nonaffected hemisphere, a number of cells shift their preferred directions to the first quadrant, because the nonaffected arm must now compensate for the movements previously performed by the affected arm (Figure 8B.3).

**Figure 9:**
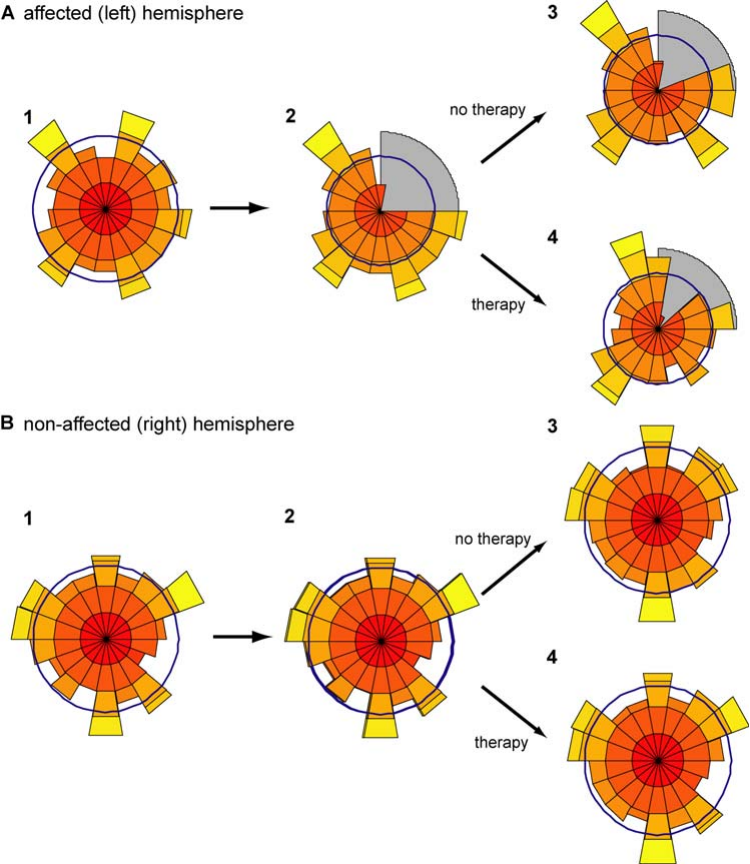
Cortical reorganization without unsupervised learning. Reorganization of the affected (left) hemisphere (A) and nonaffected (right) hemisphere (B) after stroke followed by therapy or no therapy. In each panel, histograms of the cells' preferred directions are shown (1) before stroke, (2) after stroke with 500 free choice trials, and (3) after 3,000 free choice trials or (4) after 1,000 forced used training trials and subsequent 2,000 free choice trials. The gray area in (A.2) shows the lesion site.

Without the unsupervised learning term, reorganization follows different patterns: Therapy has less of an effect on reorganization, and lack of therapy does not lead to overrepresentation of compensatory movements in the affected hemisphere or in the nonaffected hemisphere (Figure 9).

**Figure 10:**
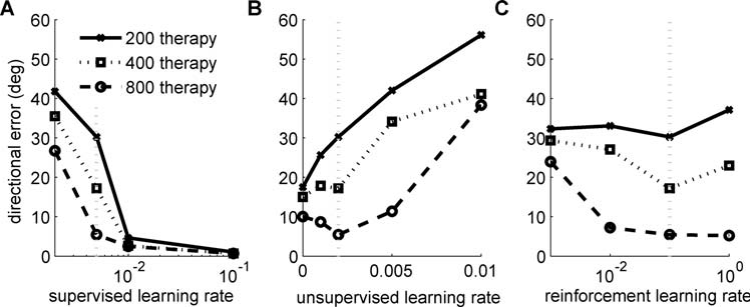
Learning rates sensitivity analysis. Effect of the supervised learning rate (A), the unsupervised learning rate (B), and the reinforcement learning rate (C) on directional error after different durations of therapy (200, 400, and 800 therapy trials) followed by 3,000 free choice condition. The default parameters used in simulations are shown with the gray vertical lines.

To better understand the respective roles of each of the supervised, unsupervised, and reinforcement learning rates on behavior we then performed a sensitivity analysis for these three parameters on directional error for different durations of therapy (200, 400 and 800 therapy trials) followed by 3,000 free choice condition. As shown in Figure 10A, directional error decreased as the supervised learning rate increased for any amount of therapy. Figure 10B shows, however, a more complex pattern for the unsupervised learning rate. For a number of rehabilitation trials sufficient to reach threshold in the default parameter set (420 therapy trials on the threshold with 0.002 for the unsupervised learning rate), there is an optimal unsupervised learning rate for which long-term performance (after 3,000 free choice trials) is enhanced compared to either zero unsupervised learning or too large unsupervised learning. Thus, for appropriate learning rates, unsupervised learning is “adaptive,” as it enhances performance. No unsupervised learning or too large unsupervised learning rates are detrimental to performance however. A similar pattern is shown for the reinforcement learning rate, although the interpretation is more arduous as very little spontaneous use occurs with a reinforcement learning rate set at 0 (to perform the sensitivity analysis for the reinforcement learning rate, we used the default parameter set until the end of the acute-stroke phase, then the different reinforcement learning rates were tested starting with therapy condition).

We further studied the conditions under which the threshold appears by setting each of the three rates to 0 and keeping the other two to the default values. With such learning rate settings, we plotted the directional error, normalized population vector, and spontaneous hand use (Figure S1, Figure S2, and Figure S3) just after therapy and 3,000 trials after therapy as a function of the number of rehabilitation trials, as in Figure 5. Unlike for the full default parameter set (Figure 5), if one of the learning rates is set to zero, the bistable behavior disappears, as shown by the noncrossing of the curves for 0 (immediate test) and 3,000 free choice trials (follow-up test). In other words, the threshold observed in the complete model is an emergent property of the three types of learning. If supervised learning or reinforcement learning is not present, directional error worsens after 3,000 free choice trials compared to just after rehabilitation, for any number of rehabilitation trials. If unsupervised learning is not present, however, directional error improves after 3,000 free choice trials for any amount of rehabilitation trials.

## Discussion

We proposed a novel model of bilateral reaching that links different levels of analysis, as it combines a simplified but biologically plausible neural model of the motor cortex, a biologically plausible (but nonneural) model of reward-based decision-making, and physical therapy intervention at the behavioral level. Because our model is based on sound theoretical principles and neural mechanisms, it allows us to explore the nonlinear interactions between performance and spontaneous use in stroke recovery.

### Cortical Reorganization after Stroke and Therapy

Our motor cortex model, by learning to minimize both directional errors and variability, accounts for the reversal of the loss of cortical representation after rehabilitation, and the increase of this loss together with the increase of the representation of neighboring areas without rehabilitation [7],[50].

In the lesioned cortex, during therapy, the supervised learning rule ensures that underrepresented directions are “repopulated,” decreasing average reaching errors. However, because there are fewer surviving neurons overall after stroke, stroke leads to a decrease in population vector magnitude (Figure 3B) and increased movement variability (Figure 7A)—as previously shown in [21]. The supervised learning component of our rule is consistent with monkey data showing that learning new skills, but not repetitive use, leads to motor cortical reorganization [51]. Supervised learning-like plasticity has not been reported in the cerebral cortex however, but it is thought to occur in the cerebellum [52]. A possibility is that the reduction of error due to rehabilitation, and the associated cortical reorganization, is driven by important cerebellar projections to the motor cortex. Lesion of the error signal driving cerebellar learning, presumably carried by the inferior olive [53], could be performed in animal models of stroke to test this possibility.

During therapy, the unsupervised learning rule is “adaptive” as its effect reinforces that of the supervised learning rule (compare Figures 8A.4 and 9A.4). By recruiting a greater number of neurons for often-performed actions it can counter neuronal noise and decrease directional error [21]; it is thus an adaptive process in the normal brain. After stroke, however, such unsupervised plasticity may become maladaptive. A comparison of Figures 8A.3 and 9A.3 shows that unsupervised learning further augments the effect of stroke if no therapy is given. As compensatory movements, or movements unaffected by the stroke, compete for the surviving neurons, fewer neurons code for directions around the affected area (Figure 8A.3), leading to further deterioration of performance (Figures 3A, 3B, and 7A). The representation of compensatory movements is increased and performance of these movements improves (Figure 7, decreased directional error bias). Without the unsupervised learning term, reorganization follows different patterns: Therapy has less of an effect on reorganization, and lack of therapy does not lead to overrepresentation of compensatory movements in the affected hemisphere or in the nonaffected hemisphere (Figure 8).

### Strengths and Limitations of the Model

To our knowledge, the present computational neural model is the first developed to make specific behavioral and neuronal predictions on the efficacy of physical therapy interventions. Two previous models have been developed to account for behavior after stroke [21],[54], but these models do not address plastic changes. The model by Goodall et al. [50] predicts that focal lesions result in a two-phase map reorganization process in the intact peri-lesion cortical region, but this model does not account for the development of compensatory movements and reorganization of choice after training.

Our model is in accord with the most recent understanding and comprehensive view of the basal ganglia function in adaptive selection of alternative actions [40],[55],[56] via release of inhibition of motor cortex activity [42]. A different decision making mechanisms was however recently proposed by Cisek [57], who analyzed the time course of cortical activation before and after decision to reach one of two targets with a single arm. Unlike in our model, target choice was resolved in a distributed manner, by competition between neurons within cortical layers. Further experiments are needed to study how targets are selected when both limbs can be used, and how this selection is reorganized after lesion and therapy.

In a recent motor cortex model [58], as in our model, reorganization of preferred directions is due to a learning rule containing two terms: a supervised error correcting term, and a (unsupervised) weight decay term. Because our unsupervised learning rule is based on the activation of neighboring neurons however, it explains maladaptation and increase of lesion size in the no-therapy condition (Figure 8A.3). Furthermore, the sensitivity analysis of the three learning rates (supervised, unsupervised and reinforcement learning, Figure 10) showed that the bistability of performance and spontaneous arm use (Figures 4 and 5) requires the combination of all three types of learning (Figures S1B, S2B, and S3B)

Because of its simplicity, our model provides clear insights into a range of factors affecting recovery of arm use after stroke. However, our model does suffer from a number of limitations:

1. The simplistic coding of the reach movements by the motor cortex neurons does not account for how activity of motor cortex neurons also correlates with joint torque and muscle activity [47],[59],[60]. The current motor cortex model was based on the directional coding of hand movement [19]. Even though a possible mechanism behind execution of directional coding on the motor cortex was set forth [61] and computational models have suggested correlation between directional coding of a neuron and a linear component the direction of force which the neuron exerted [26],[27], there is little evidence, except [45], of stroke lesions impairing specific hand directions. The key point is not the actual coding (important though directional coding undoubtedly is) but rather to see how a lesion affects a range of movements, and how learning may be maladaptive or adaptive by returning some control of that range to the unaffected or affected hand, respectively. Our assumption, how a lesion affects the distribution of neurons in the motor cortex, may be valid, only when neurons on the motor cortex form topography of directional coding. Our unpublished computational model of the motor cortex showed there exists topography of direction of population vector and this direction of force would be correlated with directional coding. Nevertheless, in the present model, as a results of such simplistic coding, directional error is highly correlated with lesion size; this may not be highly realistic as directional error after mild or moderate stroke in humans is not much affected [46].
2. A related limitation is the lack of proximal and distal representation in our motor cortex model. In the biological motor cortex, individual joints are controlled by somewhat overlapping neural groupings forming somatotopically organized and plastic motor cortical maps. Empirical results of map reorganization after lesion have focused on remapping of the hand region [7],[8]. It is to be noted however, that although our model focuses on redistribution of the representation of reaching directions within the area of cortex, our results accord well with the type of reorganization shown in these empirical results.
3. A third limitation is our simplistic model of stroke, akin to that used in animal models of stroke. These ignore the motor impairments due to diffuse lesions to a number of brain areas and tracts, and not just to the motor cortex. In particular, our model cannot study the differential effect of cortical, subcortical and combined cortical–subcortical strokes and thus cannot account for differential response to rehabilitation for different stroke locations (e.g., [62]).

To resolve the limitations, in the future we will expand our model by adding arm and muscle models controlled by neurons grouped in adaptive motor cortical maps. We plan to investigate the tradeoff between proximal and distal regions, with cortical motor maps that change during training on tasks that require more skilled use of the hand itself. Moreover, the notion that the action choice model may correspond to the basal ganglia opens up promising lines of investigation.

In summary, despite our considerable simplifications of movement representation in the motor cortex and of the simulated lesions, our results show that our proposed mechanism of motor learning and plasticity, and the ensuing results (recovery, threshold, and neural reorganization) are general and not particular to the specifics of our model.

### Specific and Testable Predictions Derived from the Model

Our model makes the following testable behavioral and neural predictions.

#### Prediction 1

If spontaneous use of the affected arm is above a threshold level after therapy, repeated spontaneous attempts to use the affected arm leads to further improvements in motor performance, which in turn increase the “value” of using the arm (Figure 3).

#### Prediction 2

If spontaneous arm use is below this threshold after therapy, compensatory movements are reinforced. Consequently, spontaneous use and motor performance of the affected limb decrease (Figure 3).

#### Prediction 3

The dose of task practice necessary to reach the threshold depends on stroke severity, and no amount of rehabilitation will be sufficient to reach this threshold for most strokes that are classified as severe (Figures 4 and 6).

#### Prediction 4

Unless the stroke impairment is too severe, the dose o f rehabilitation can be adjusted for each patient such that spontaneous arm use reaches this critical threshold after rehabilitation. If the stroke is too severe however, motor retraining is “in vain” (Figures 4 and 6). Of course, the dose of task practice also depends on parameters within the model, and these may represent intersubject variability of stroke patients that complements the effects of lesion size.

#### Prediction 5

After effective motor retraining, movement accuracy can return close to its prestroke levels, but movement variability will be higher than prestroke (Figure 7)

#### Prediction 6

After noneffective retraining, compensatory movements, either with the same limb or the other limb or both, will become less variable (Figure 7).

#### Prediction 7

The hemisphere contralateral to the lesion undergoes reorganization of preferred reach directions along with the development of compensatory reach movements in the affected range (Figure 8).

#### Prediction 8

Both supervised learning-like (error driven) and unsupervised learning-like (use driven) plastic phenomena drive reorganization in the motor cortex during skill learning in the normal brain and after stroke (Figures 8 and 9).

### Implication for Rehabilitation

In our model, neural reorganization generates bistability at the behavioral level: after therapy, spontaneous arm use will stabilize at either a low or a high value, depending on the amount of therapy. Specifically, therapy is effective and could be stopped if spontaneous arm use reaches a certain threshold, as the repeated spontaneous arm use following therapy provides a form of motor learning that further “bootstraps” performance. Below this threshold, however, motor retraining is “in vain”—there is no or little long-term spontaneous arm use after training, and the model exhibit “learned nonuse,” as has been proposed in patients with brain lesions [13].

We thus predict that a measure of spontaneous arm use may be a good indicator to determine optimal duration of the therapy. In current rehabilitation practice, all rehabilitation is concentrated in the weeks following stroke. Our model suggests that rehabilitation protocols adopt instead a spaced and adaptive train–Test A–wait–test B–train paradigm: short bouts of training (train) are followed by a spontaneous arm use test (Test A), no training for several weeks (wait), and another spontaneous arm use tests (Test B). If spontaneous arm use measured on Test B has increased since that on test Test A, the threshold is reached, and rehabilitation can be terminated. If spontaneous arm use is still low or has decreased since Test A, another bout of rehabilitation is called for. This pattern is repeated until the threshold is reached. Note that such a training paradigm will have the additional benefit of making use of the “spacing effect,” in which spaced training lead to superior retention of learned skills [63]. We plan to put this hypothesis to empirical test using a novel laboratory-based objective test of bilateral limb use.

## Supporting Information

Text S1 Supplemental materials: Learning rule derivation.
(0.40 MB DOC)

Figure S1 Effect of supervised learning. (A) Directional error, (B) normalized population vector (PV), (C) and spontaneous arm use after different durations of therapy followed by 0 free choice trial (immediate) and 3000 free choice trials (follow-up) without supervised learning. Unlike in the full model (see Figure 5), the bistable behavior is not present, as shown by the non-crossing of the curves in the immediate and follow-up condition.
(0.17 MB TIF)

Figure S2 Effect of unsupervised learning. (A) Directional error, (B) normalized population vector (PV), and (C) spontaneous arm use after different durations of therapy followed by 0 free choice trial (immediate) and 3000 free choice trials (follow-up) without unsupervised learning. Unlike in the full model (see Figure 5), the bistable behavior is not present, as shown by the non-crossing of the curves in the immediate and follow-up condition.
(0.18 MB TIF)

Figure S3 Effect of reinforcement learning. (A) Directional error, (B) normalized population vector (PV), and (C) spontaneous arm use after different durations of therapy followed by 0 free choice trial (immediate) and 3000 free choice trials (follow-up) without reinforcement learning. Unlike in the full model (see Figure 5), the bistable behavior is not present, as shown by the non-crossing of the curves in the immediate and follow-up condition. In these simulations, we first used a positive reinforcement learning rate (0.01) during acute stroke phase (500 free choice trials after lesion), before “turning off” reinforcement learning in the following trials. Due to supervised learning and unsupervised learning, performance improved over time but spontaneous arm use stayed low.
(0.17 MB TIF)
