# Supplementary material for: Stroke Rehabilitation Reaches a Threshold
Source: PLoS Comput Biol. 2008 Aug 22;4(8):e1000133. doi: 10.1371/journal.pcbi.1000133 (PMC2527783; doi:10.1371/journal.pcbi.1000133)
Supplement: Text S1 — Supplemental materials: Learning rule derivation. (0.40 MB DOC) [file pcbi.1000133.s001.doc]

# Supplemental Materials

**A. Learning rule derivation**

The partial derivatives of the cost function in equation (2) with respect to the preferred direction of the ith neuron, *θ_p_^i^* .

$\frac{\delta E}{\delta\theta_{p}^{i}}=\left\{\begin{matrix} (\theta_{e}-\theta_{d})\frac{\delta\theta_{e}}{\delta\theta_{p}^{i}}-\lambda'\cos(\theta_{d}-\theta_{p}^{i})\sin(\theta_{d}-\theta_{p}^{i})\text{ }\left|\theta_{d}-\theta_{p}^{i}\right|<\frac{\pi}{2} \\ (\theta_{e}-\theta_{d})\frac{\delta\theta_{e}}{\delta\theta_{p}^{i}}-0\text{ }\left|\theta_{d}-\theta_{p}^{i}\right|\geq\frac{\pi}{2} \end{matrix}\right.$ (A.1)

where $\lambda'$ =$2\lambda$. This equation is valid where $\left|\theta_{d}-\theta_{p}^{i}\right|<\pi/2$, because y is differentiable in this range; otherwise the second term equals zero.

*Derivation of the first term in the right hand side of equation (A.1)*

We used a geometrical approximation to $\frac{\delta\theta_{e}}{\delta\theta_{p}^{i}}$(Figure S.A).


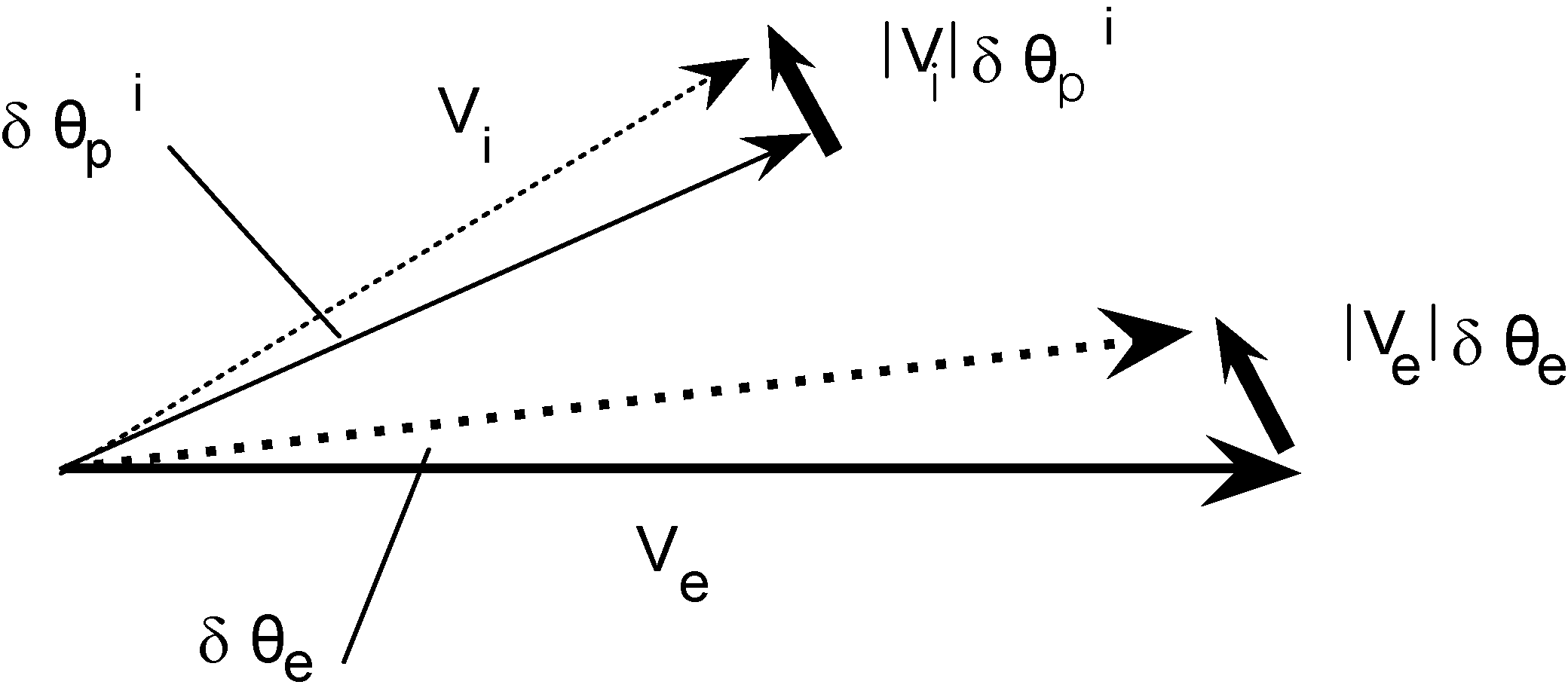


Figure S.A. Approximation of $\frac{\delta\theta_{e}}{\delta\theta_{p}^{i}}$. V_i_ is a vector for preferred direction $\theta_{p}^{i}$ , and V_e_ a vector for the executed direction $\theta_{e}$.

A perturbation on the preferred direction $\delta\theta_{p}^{i}$also perturbed the cell’s vector V_i_ along this direction of magnitude $y^{i}$. This perturbation in the vector also perturbed the population vector V_e_ on the executed direction. Because the amount of perturbation in the specific individual vector, $|V_{i}|\delta\theta_{p}^{i}$ and the amount of perturbation in the population vector, $|V_{e}|\delta\theta_{e}$ are equal,

$|V_{i}|\delta\theta_{p}^{i}=|V_{e}|\delta\theta_{e}\Leftrightarrow\frac{\delta\theta_{e}}{\delta\theta_{p}^{i}}=\frac{|V_{i}|}{|V_{e}|}=\frac{1}{|V_{e}|}y^{i}$ (A.2)

Thus we approximate $\frac{\delta\theta_{e}}{\delta\theta_{p}^{i}}$with $y^{i}$, the only available local (and thus biologically plausible) information. $\frac{1}{|V_{e}|}$ is a scaling factor and can be absorbed into the learning rate of supervised learning.

We can then obtain the supervised learning rule in equation (2).

$(\theta_{e}-\theta_{d})\frac{\delta\theta_{e}}{\delta\theta_{p}^{i}}\approx(\theta_{e}-\theta_{d})\frac{y^{i}}{|V_{e}|}\text{ }\left(∵\frac{\delta\theta_{e}}{\delta\theta_{p}^{i}}\approx\frac{y^{i}}{|V_{e}|}\right)$ (A,3)

We verified both analytically and in simulations that the approximation $\frac{\delta\theta_{e}}{\delta\theta_{p}^{i}}\approx\frac{y^{i}}{|V_{e}|}$ is appropriate (please contact the corresponding author to obtain the exact derivation and corresponding simulation results).

*Derivation of the second term in the right hand side of equation (A.1)*

The second term can be approximated with

$\begin{matrix} \lambda'\cos(\theta_{d}-\theta_{p}^{i})\sin(\theta_{d}-\theta_{p}^{i})\approx\lambda'y^{i}\text{ }(\theta_{d}-\theta_{p}^{i})\text{ } \\ \text{ }(∵y^{i}=\cos(\theta_{d}-\theta_{p}^{i}),\sin(x)\approx x) \end{matrix}$ (A.4)

Note that equation (A.4) is valid where $\left|\theta_{d}-\theta_{p}^{i}\right|<\pi/2$; otherwise this term is equal to zero. The first Taylor expansion of sine function is valid for $\theta_{p}^{i}$ ≈$\theta_{d}$. However, due to truncation of the neurons’ activation rule (Equation (1)), if $\theta_{p}^{i}$ is far from $\theta_{d}$, where the approximation of the sine function is invalid, $y^{i}$ is zero or near zero. Thus, this approximation is valid for all directions. We verified in simulations, that compared to a non-approximated equation, the maximum error is about 13%.

In summary, the weight update rule is

$\begin{matrix} \theta_{p}^{i}\leftarrow\theta_{p}^{i}-\alpha\frac{\delta E}{\delta\theta_{p}^{i}}\approx\theta_{p}^{i}-\alpha(\theta_{e}-\theta_{d})\frac{y^{i}}{|V_{e}|}+\alpha\lambda'(\theta_{d}-\theta_{p}^{i})y^{i} \\ \text{ }=\theta_{p}^{i}+\alpha_{SL}(\theta_{d}-\theta_{e})y^{i}+\alpha_{UL}(\theta_{d}-\theta_{p}^{i})y^{i} \end{matrix}$ (A.5)

with $\alpha$≥ 0, $\alpha_{SL}$ = $\frac{\alpha}{|V_{e}|}$ the learning rate of the supervised learning rule, and $\alpha_{UL}$.= $\alpha\lambda'$ the learning rate of the unsupervised learning rule.

**B. Effect of each learning process on bistability**


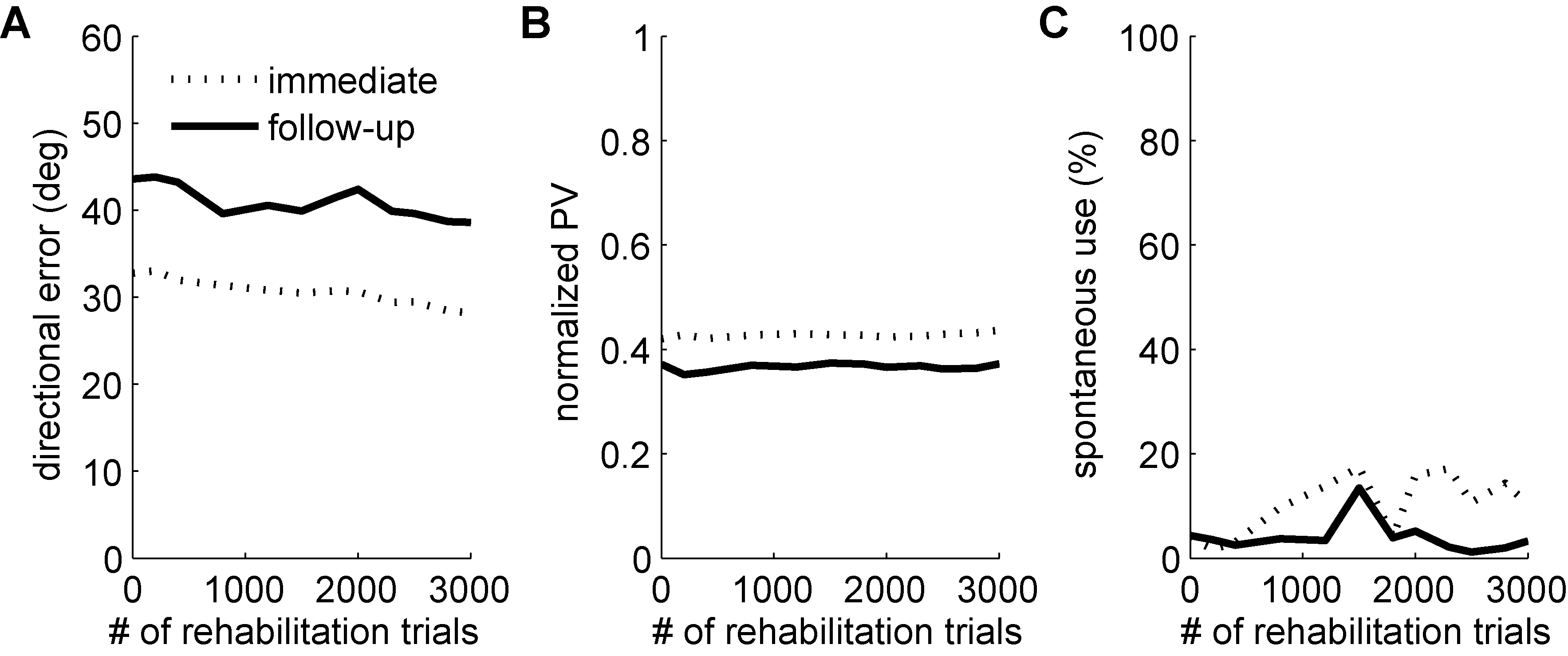


Figure S1. Effects of supervised learning. (A) Directional error, (B) normalized population vector (PV), (C) and spontaneous arm use after different durations of therapy followed by 0 free choice trial (immediate) and 3000 free choice trials (follow-up) without supervised learning. Unlike in the full model (see Figure 5), the bistable behavior is not present, as shown by the non-crossing of the curves in the immediate and follow-up condition.


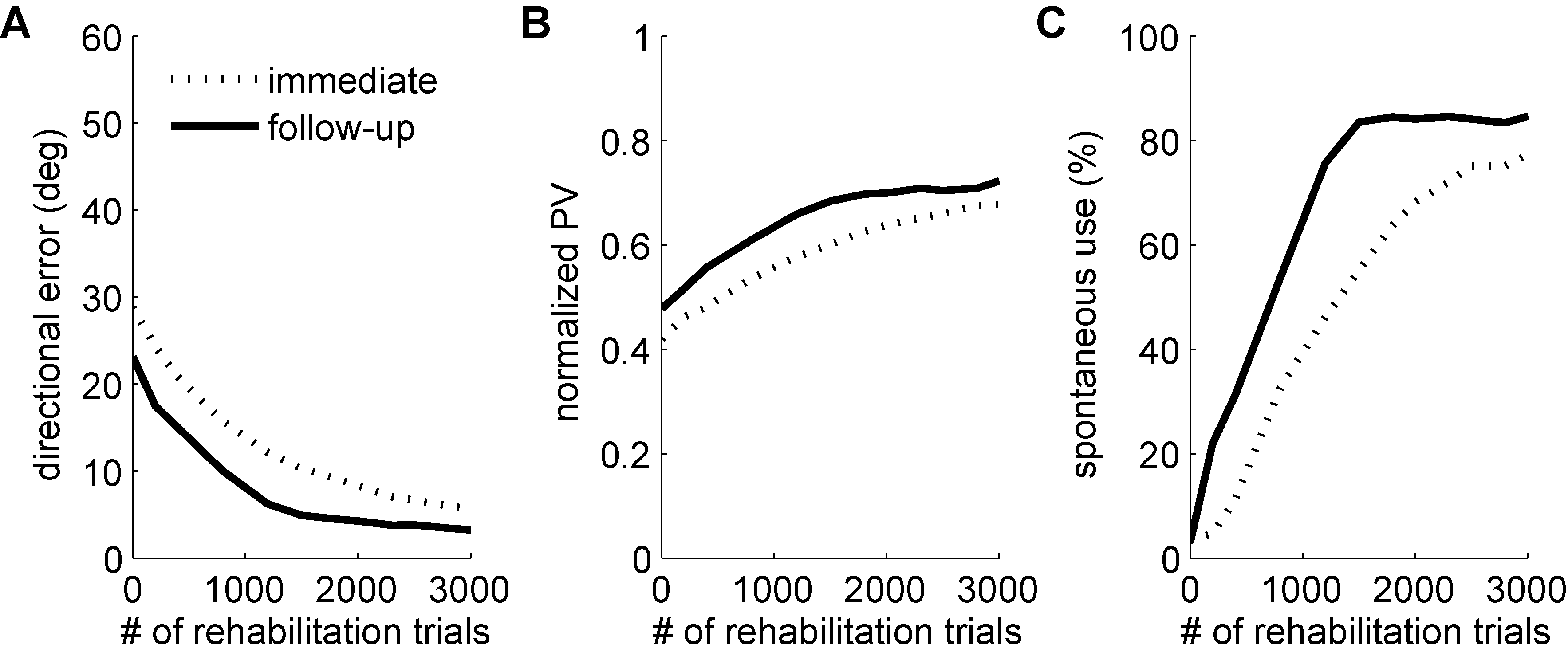


Figure S2. Effects of unsupervised learning. (A) Directional error, (B) normalized population vector (PV), and (C) spontaneous arm use after different durations of therapy followed by 0 free choice trial (immediate) and 3000 free choice trials (follow-up) without unsupervised learning. Unlike in the full model (see Figure 5), the bistable behavior is not present, as shown by the non-crossing of the curves in the immediate and follow-up condition.


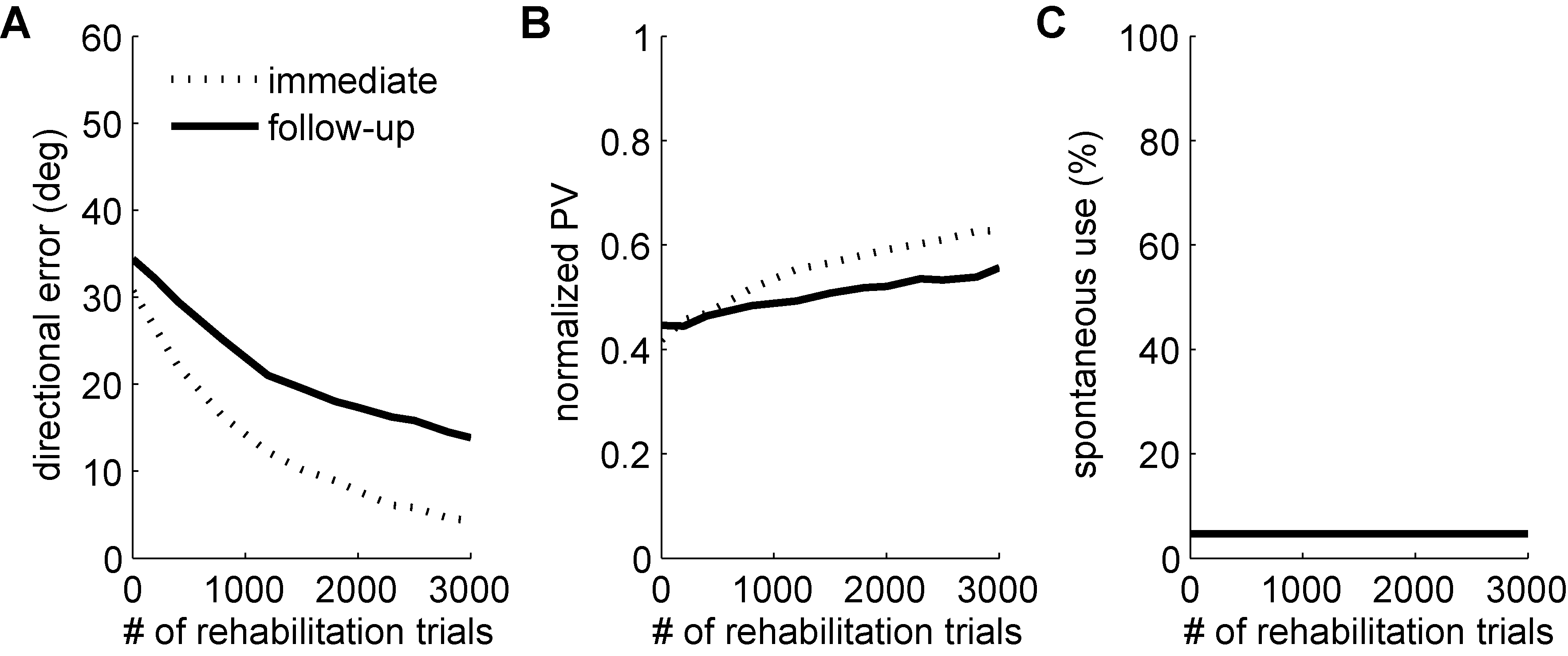


Figure S3. Effects of reinforcement learning. (A) Directional error, (B) normalized population vector (PV), and (C) spontaneous arm use after different durations of therapy followed by 0 free choice trial (immediate) and 3000 free choice trials (follow-up) without reinforcement learning. Unlike in the full model (see Figure 5), the bistable behavior is not present, as shown by the non-crossing of the curves in the immediate and follow-up condition. In these simulations, we first used a positive reinforcement learning rate (0.01) during acute stroke phase (500 free choice trials after lesion), before “turning off” reinforcement learning in the following trials. Due to supervised learning and unsupervised learning, performance improved over time but spontaneous arm use stayed low.
