# Supplementary figures and images for: Stroke Rehabilitation Reaches a Threshold
Source: PLoS Comput Biol. 2008 Aug 22;4(8):e1000133. doi: 10.1371/journal.pcbi.1000133 (PMC2527783; doi:10.1371/journal.pcbi.1000133)

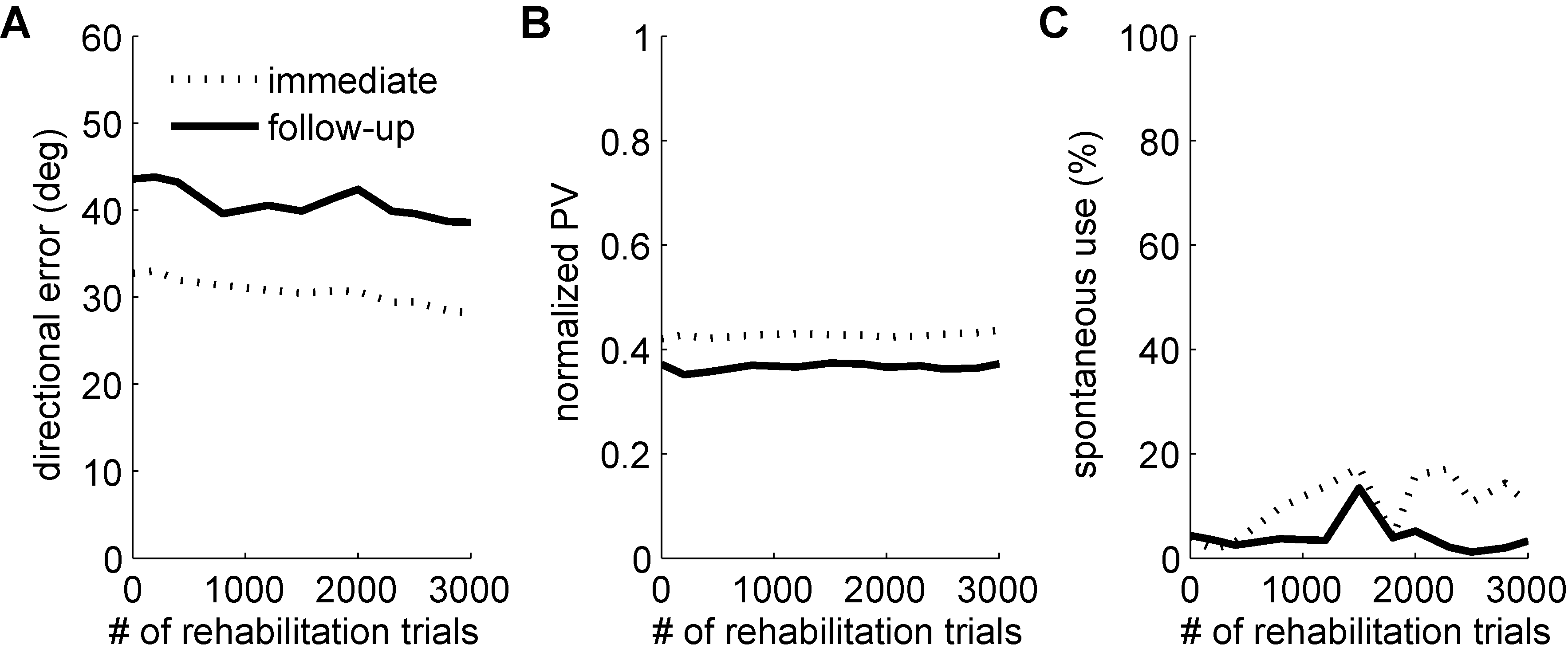

Supplement: Figure S1 — Effect of supervised learning. (A) Directional error, (B) normalized population vector (PV), (C) and spontaneous arm use after different durations of therapy followed by 0 free choice trial (immediate) and 3000 free choice trials (follow-up) without supervised learning. Unlike in the full model (see Figure 5), the bistable behavior is not present, as shown by the non-crossing of the curves in the immediate and follow-up condition. (0.17 MB TIF) [file pcbi.1000133.s002.tif]

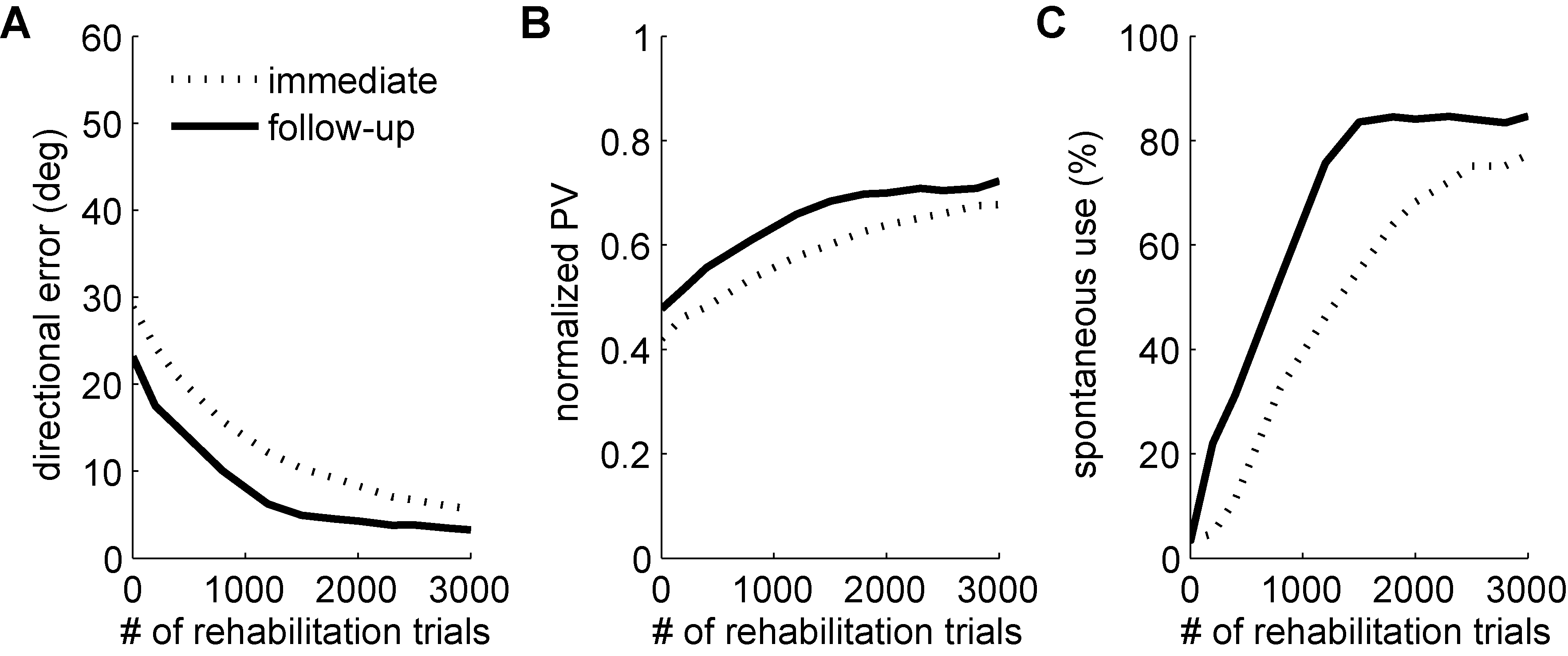

Supplement: Figure S2 — Effect of unsupervised learning. (A) Directional error, (B) normalized population vector (PV), and (C) spontaneous arm use after different durations of therapy followed by 0 free choice trial (immediate) and 3000 free choice trials (follow-up) without unsupervised learning. Unlike in the full model (see Figure 5), the bistable behavior is not present, as shown by the non-crossing of the curves in the immediate and follow-up condition. (0.18 MB TIF) [file pcbi.1000133.s003.tif]

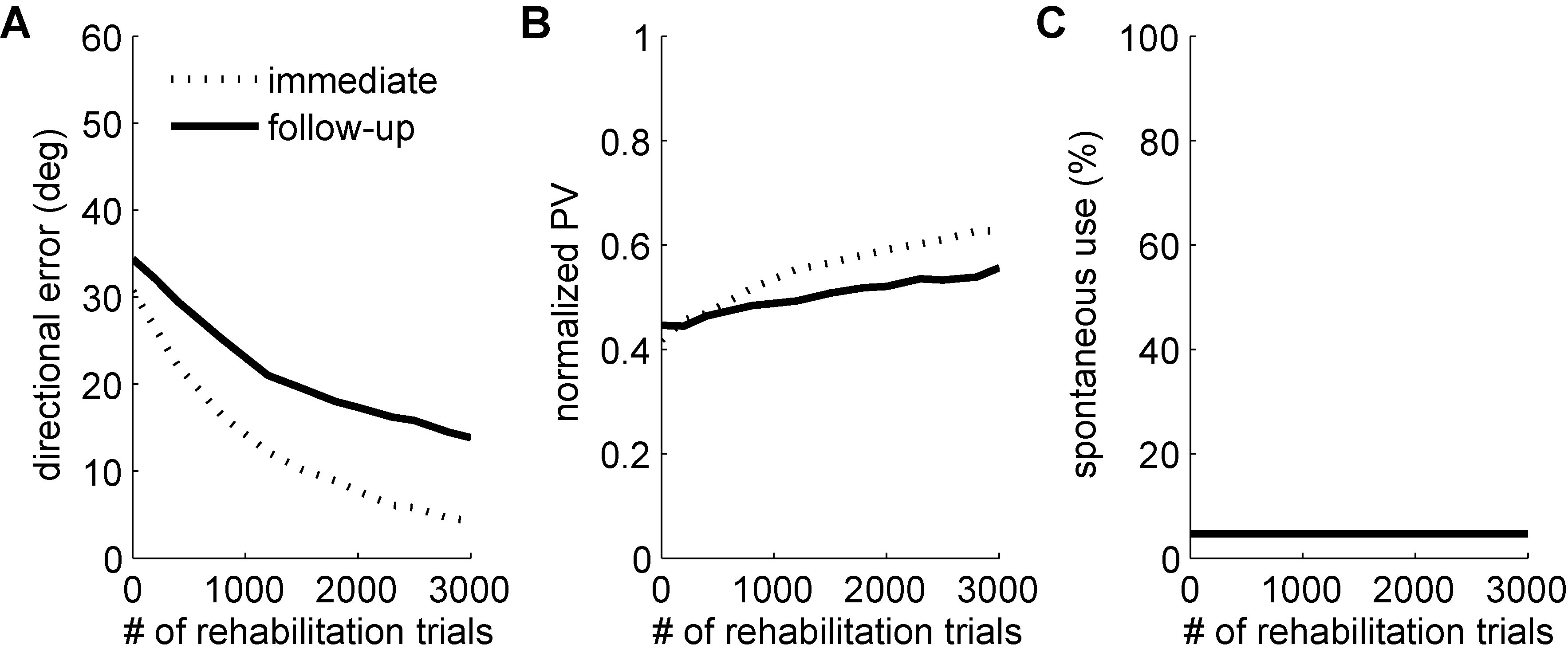

Supplement: Figure S3 — Effect of reinforcement learning. (A) Directional error, (B) normalized population vector (PV), and (C) spontaneous arm use after different durations of therapy followed by 0 free choice trial (immediate) and 3000 free choice trials (follow-up) without reinforcement learning. Unlike in the full model (see Figure 5), the bistable behavior is not present, as shown by the non-crossing of the curves in the immediate and follow-up condition. In these simulations, we first used a positive reinforcement learning rate (0.01) during acute stroke phase (500 free choice trials after lesion), before “turning off” reinforcement learning in the following trials. Due to supervised learning and unsupervised learning, performance improved over time but spontaneous arm use stayed low. (0.17 MB TIF) [file pcbi.1000133.s004.tif]
